# A Phase II Study of Stereotactic Body Radiation Therapy for Low-Intermediate-High-Risk Prostate Cancer Using Helical Tomotherapy: Dose-Volumetric Parameters Predicting Early Toxicity

**DOI:** 10.3389/fonc.2014.00336

**Published:** 2014-11-26

**Authors:** Victor A. Macias, Manuel L. Blanco, Inmaculada Barrera, Rafael Garcia

**Affiliations:** ^1^Radiation Oncology Department, Salamanca University Hospital, Salamanca, Spain; ^2^Department of Statistics, University of Salamanca, Salamanca, Spain; ^3^Radiation Oncology Department, CyberKnife Unit, IMO Group, Madrid, Spain

**Keywords:** prostate cancer, stereotactic body radiation therapy, tomotherapy, predictors toxicity, common toxicity criteria, IPSS

## Abstract

**Endpoint:** To assess early urinary (GU) and rectal (GI) toxicities after helical tomotherapy Stereotactic body radiation therapy (SBRT), and to determine their predictive factors.

**Methods:** Since May 2012, 45 prostate cancer patients were treated with eight fractions of 5.48 (low risk, 29%) or 5.65 Gy (intermediate-high risk, 71%) on alternative days over 2.5 weeks. The exclusion criteria were Gleason score 9–10, PSA >40 ng/mL, cT3b-4, IPSS ≥20, and history of acute urinary retention. During the follow-up, a set of potential prognostic factors was correlated with urinary or rectal toxicity.

**Results:** The median follow-up was 13.8 months (2–25 months). There were no grade ≥3 toxicities. Acute grade 2 GU complications were found in a 22.7% of men, but in 2.3% of patients at 1 month, 0% at 6 months, and 0% at 12 months. The correspondent figures for grade 2 GI toxicities were 20.4% (acute), 2.3% (1 month), 3.6% (6 months), and 5% (12 months). Acute GI toxicity was significantly correlated with the rectal volume (>15 cm^3^) receiving 28 Gy, only when expressed as absolute volume. The age (>72 years old) was a predictor of GI toxicity after 1 month of treatment. No correlation was found, however, between urinary toxicity and the other analyzed variables. IPSS increased significantly at the time of the last fraction and within the first month, returning to the baseline at sixth month. Urinary-related quality of life (IPSS question 8 score), it was not significantly worsen during radiotherapy returning to the baseline levels 1 month after the treatment. At 12 months follow-up patient’s perception of their urinary function improved significantly in comparison with the baseline.

**Conclusion:** Our scheme of eight fractions on alternative days delivered using helical tomotherapy is well tolerated. We recommend using actual volume instead of percentual volume in the treatment planning, and not to exceed 15 cm^3^ of rectal volume receiving ≥25 Gy in order to diminish acute GI toxicity.

## Introduction

The Canadian Association of Radiation Oncology defines stereotactic body radiation therapy (SBRT) as the precise delivery of highly conformal and image-guided hypofractionated external-beam radiotherapy, delivered in a single or few fraction(s) to an extracraneal body target with doses at least biologically equivalent to a radical course when given over a protracted conventionally (1.8–3.0 Gy/fraction) fractionated schedule (1). The American Society for Radiation Oncology has recently stated that “data supporting the use of SBRT for prostate cancer have matured to a point where SBRT could be considered an appropriate alternative for selective patients with low to intermediate-risk disease.” There are not yet available data from controlled randomized trials comparing SBRT with moderate hypofractionated or normofractionated radiation therapy. Most of the SBRT studies are phase II prospective trials using a dedicated robotic linear accelerator (Cyberknife, Accuracy, Sunnyvale, CA, USA) (2–11). The most common prescriptive dose is five fractions of 7–7.5 Gy (12). Given the prostate cancer radiobiology, tumor cells receive biologically equivalent doses as high as 85–91 Gy (α/β 1.5), which ensures a biochemical recurrence free survival over 90% at 3–5 years for low and intermediate risk. Acute and late grade ≥3 events are rare in these selected studies. Recently, a few phase II trials on prostate SBRT using non-robotic linear accelerators have been published with limited numbers of patients and short follow up. There is a limited experience on this approach (13–17). The theoretical advantages are that the homogeneous dose distribution and the slightly protracted scheme may decrease toxicity. We are reporting an analysis of the predictors for early toxicity after an 8-fraction regimen on alternative days delivered by helical tomotherapy in low-intermediate-high-risk prostate cancer patients.

## Materials and Methods

### Patients

This is a single institution phase II trial study approved in 2012 by the Salamanca University Hospital Institutional Review Board. The main purpose was to study early and late side effects of SBRT hypofractionated radiotherapy for prostate cancer with helical tomotherapy. It was designed to enroll 107 patients, assuming a loss of 10%.

The selection of patients, treatment planning, and delivery were described in a previous article (18). Briefly, patients selected to be included in the study had histologically confirmed adenocarcinoma of the prostate cT1-3a, Gleason score <8, and initial PSA <20 ng/mL. Since May 2013 patients over 70 years old with Gleason score 8 and PSA <10 ng/mL or PSA 20–40 ng/mL and Gleason score ≤7 were also selected. Exclusion criteria were clinical stage cT3b-4, involved lymph nodes or distant metastases on imaging, prior pelvic radiotherapy, International Prostate Symptom Score System (IPSS) >20, or history of acute urinary retention. Patients were regrouped by version 3.2012 NCCN guidelines (low risk: PSA <10 and Gleason sum of 6 and clinical stage T1c–T2a, intermediate risk: PSA 10–20 or Gleason sum of 7 or clinical stage T2b-c, high-risk: PSA >20 or Gleason sum 8–10 or clinical stage T3a).

### Simulation and treatment planning

**Figure 1:**
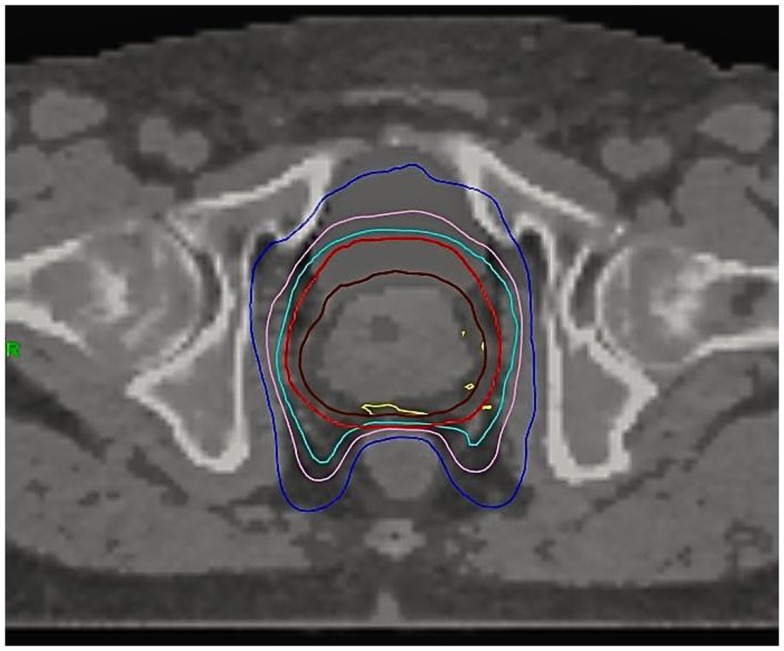
**Dose distribution on a typical patient**. CTV (brown), PTV (red), 95% isodose (light blue), 37 Gy isodose (pink), 28 Gy isodose (dark blue), 103% isodose (yellow).

An anti-flatulence diet and mild oral laxatives were taken 2 weeks before simulation CT until the end of radiotherapy treatment. Bladder was filled with 250 ml contrast material through a urinary catheter at the time of the simulation CT. Intraprostatic fiducials were not implanted. The clinical target volume (CTV) included the prostate and the proximal seminal vesicles (at the point where the seminal vesicles separate). A contour was drawn around the prostate gland with margins of about 2–5 mm, depending on the prostate cancer risk group (19, 20). CTV to PTV margins were based on recommendations of two series with image guidance with implanted fiducials (21, 22). CTV was expanded 6 mm in the craniocaudal direction, 3 mm posteriorly, 4–5 mm laterally, and anteriorly up to symphysis pubis (about 7–10 mm). The rectum was contoured as a solid organ up to 1 cm above and below the PTV-containing sections. The posterior half of the rectum was also contoured on each CT slice (23). Not more than the 2% of the volume should receive ≥37 Gy. This auxiliary volume was created to help make a sharp dose gradient on the anterior rectum. Figure 1 shows the dose distribution on a typical patient. The prescription dose was 43.84 or 45.2 Gy to the PTV delivered in eight fractions on alternative days, which corresponds to a tumor equivalent dose at 2 Gy/fraction (EQD2) of approximately 87.4 or 92.3 Gy for NCCN low risk and intermediate-high risk, respectively, assuming a α/β ratio of 1.5 Gy. Correspondent figures for late-responding normal tissues (α/β of 3 Gy) are 74.3 and 78.2 Gy, respectively. Dose was prescribed to 95% of PTV. All cases were contoured, reviewed, and approved by a single physician (Victor A. Macias).

### Treatment delivery

Megavoltage CT images (MVCT) were acquired before each fraction using the on-board scanner of the Tomotherapy unit and were co-registered to the simulation CT using automatic fusion of bony anatomy and soft tissue. The registration was further manually adjusted to account for inter-fractional motion. We ensured that the prostate was within the 95% isodose, minimizing at the same time the volume of rectum irradiated. Endorectal balloons were not used. Patients measured the volume they urinated just after each treatment fraction in order to have feedback information to help keep their bladder volume between 200 and 300 cm^3^. The median of the eight measurements was also recorded (MVU). During radiotherapy, the patients were prescribed with 0.4 mg tamsulosin and corticosteroid enema (fluocinilone).

### Hormonal therapy

Intermediate-high-risk patients were prescribed with an androgen deprivation therapy (AD) before being sent to Radiation Oncology, which consisted of one injection of 6-month gonadotropin-releasing hormone agonist plus 50 mg/day bicalutamide for the first month. AD was, therefore, neoadjuvant and concomitant to radiation therapy.

### Follow-up

Acute toxicity collects complications during radiotherapy and the following 2 weeks. Toxicity was prospectively documented at follow-up visits at month 1, 6, 12, 18, and 24 after the completion of SBRT using the common terminology criteria for adverse events (CTCAE) version 4.0. PSA and total testosterone were obtained during the visits. IPSS questionnaires were filled out by patients before radiotherapy, at the last fraction and during the follow-up visits. IPSS question eight (Q8) refers to the patient’s perceived urinary-related quality of life. Biochemical failure was defined as a rise of 2 ng/mL over nadir.

### Statistical analysis

All statistical analyses were performed with IBM SPSS software version 21.0 and some graphics were made with Microsoft Excel version 14.3.9 for Mac. The following variables were studied as potential prognostic factors of acute toxicity: age, Charlson Comorbidity Index, risk group, AD, total dose, basal IPSS, CTV, PTV, rectal volume, bladder volume, and the median of the volume of urine measured immediately after each of the eight fractions (MVU) and finally, the amount of rectum or bladder (absolute or percentage volume), which receives the dose levels used in the treatment planning (≥43, ≥40, ≥37, ≥34, and ≥28 Gy). These variables were also correlated with urinary or rectal toxicity during follow up. Sample medians and ranges were used to describe continuous variables, except age, whose mean and SD was used instead. For qualitative variables, frequencies, and/or percentages were used. It was examined whether the variables were correlated with urinary or rectal toxicity along the follow-up. Student’s *t*-test or ANOVA test were used with age, while U-Mann Whitney, or Kruskal–Wallis tests were used with the other quantitative variables. Receiver operating characteristic curve (ROC curve) analysis provided us the statistical methods to determine the cut-off value for a variable with the highest sensitivity and specificity in order to classify patients without toxicity versus with toxicity. The chi-square test was used with qualitative variables. Student’ s *t*-test and Wilcoxon test were used to assess differences in ongoing PSA and quality of life scores (total IPSS score and Q8) in comparison to the baseline. As reported by Chen et al. (24), the minimally important difference (MID) in IPSS score was defined as a change of one-half SD from the baseline.

## Results

### Description of our series

In the period starting on May 2012 up to April 2014, 45 prostate cancer patients were treated following the SBRT protocol at the Salamanca University Hospital. Table 1 shows patient characteristics. By NCCN classification, 71% belonged to the intermediate or high-risk group. Eight of these were elderly men with Gleason score 8 and PSA <10 ng/mL or PSA 20–40 ng/mL and Gleason score ≤7. Most patients (80%) had a Charlson Comorbidity Index of 0–1. Thirty-two patients received 45.2 Gy at 5.65 Gy/fraction and 13 patients received 43.84 Gy at 5.48 Gy/fraction. Plans were homogeneous by design, with doses ranking from 95 to 103% within the PTV. The 98% isodose covered at least 95% of the PTV (median 99.2% of the PTV). A summary of the dose-volume histogram is shown in Table 2. The median volume of MVU is 260 ml (range 125–400), suggesting bladder volume through the course of radiotherapy is not very different from the bladder volume contoured on the simulation CT.

**Table 1:** **Patient, tumor, and treatment characteristics**.

Age	Mean ± SD (years)	70.40 ± 7.19
Charlson comorbidity index	0	26∕45(57.8%)
	1	10∕45 (22.2%)
	2	8∕45 (17.8%)
	4	1∕45 (2.2%)
IPSS pre-RT	Median (range)	5 (0–14)
	0–4	17∕45 (37.8%)
	5–9	16∕45 (35.6%)
	10–14	11∕45 (24.4%)
	NA	1∕45 (2.2%)
PSA	Median (ng/mL)	9
	<10	25∕45 (55.6%)
	10–20	17∕45 (37.6%)
	>20	3∕45 (6.7%)
cT stage	1c	19∕45 (42.2%)
	2a	4∕45 (8.9%)
	2b	6∕45 (13.3%)
	2c	2∕45 (6.7%)
	3a	13∕45 (28.9%)
Pelvic MRI	Yes	16∕45 (35.6%)
	No	29∕45 (64.4%)
Gleason score	5	1∕45 (2.2%)
	6	22∕45 (48.9%)
	7	17∕45 (37.8%)
	8	5∕45 (11.1%)
NCCN risk group	Low	13∕45 (28.9%)
	Intermediate	17∕45 (37.8%)
	High	15∕45 (33.3%)
Androgen deprivation (AD)	Yes	35∕45 (77.8%)
	No	10∕45 (22.2%)
Total dose (Gy)	Median	45.2
Dose/fraction (Gy)	Median	5.65
Irradiation time (s)	Median (range)	513 (384–695)
AD duration (months)	Median (range)	6 (6–26)

**Table 2:** **Summary of dose-volume histogram data**.

	Median	Min	Max
CTV (cm^3^)	113.70	53.00	209.90
PTV (cm^3^)	201.0	61.20	339.70
Rectum (cm^3^)	43.60	24.70	89.00
Bladder (cm^3^)	224.00	19.50	346.60
Penile bulb (cm^3^)	6.70	3.60	13.00
PTV V98% (%)	99.15	95.00	100.00
PTV D2% (Gy)	46.40	44.20	47.30
Rectum V43Gy (cm^3^)	2.7	0.1	12.70
Rectum V40Gy (cm^3^)	5.25	1.20	71.40
Rectum V37Gy (cm^3^)	7.45	3.60	22.60
Rectum V34Gy (cm^3^)	9.6	4.9	28.3
Rectum V28Gy (cm^3^)	14.65	8.3	45
Bladder V43Gy (%)	15.5	8.00	26.70
Bladder V40 Gy (%)	20.20	10.30	36.90
Bladder V37Gy (%)	25.40	13.50	46.20

The median follow-up is 13.8 months (range 2–25 months). All patients but one were able to complete the treatment. That patient died in an accident after the sixth fraction. Thirty-five patients received neoadjuvant-concomitant AD for 6–26 months (median 6 months). The eight elderly high-risk selected patients above mentioned, also received neoadjuvant-concomitant AD, instead of the neoadjuvant-concomitant-adjuvant AD for 2–3 years, which is the standard for high-risk patients in our institution. Six out of 13 low-risk patients were prescribed AD before being sent to Radiation Oncology.

### Biochemical failure

So far, there is one intermediate-risk patient that met the biochemical failure definition at 17 months post-treatment. PSA rose to 3.34 ng/mL after a nadir level of 0.44 ng/mL, however, it fell to 2.09 ng/mL 2 months later. The upcoming PSA tests will serve to distinguish true biochemical recurrence from a PSA bounce. For the whole series, pre-treatment total PSA levels ranged from 1.2 to 34 ng/mL with a median value of 9 ng/mL. At 1 month after SBRT, the median PSA value was 0.1 ng/mL (0.010–9.800) with a median total serum testosterone level of 19 ng/dL, while at 12 months after radiotherapy the correspondent figures were 0.16 ng/mL (0.002–3.000) and 200 ng/dL.

### Toxicity

No grade 3 or 4 toxicities were encountered. Maximum urinary and rectal complications are reported in Table 3. The acute urinary toxicities were dysuria (18 grade 1 and 5 grade 2), nocturia (13 grade 1 and 3 grade 2), urinary frequency (9 grade 1 and 3 grade 2), retention (4 grade 1 and 2 grade 2, one of which had acute retention), and finally urgency (3 grade 1 and 2 grade 2). The acute intestinal complications were proctitis/hemorrhoidal pain during bowel movements (8 grade 1, 2 grade 2), frequency with normal stool consistency (7 grade 1, 1 grade 2), bleeding (7 grade 1, 2 grade 2), tenesmus (3 grade 1, 4 grade 2), incontinence (4 grade 1, 1 grade 2), abdominal pain (5 grade 1), and diarrhea (2 grade 1 and 1 grade 2).

**Table 3:** **Prevalence of urinary and intestinal toxicities at each follow-up**.

	Urinary toxicity				Intestinal toxicity			
	Grade 0	Grade 1	Grade 2	Grade 3	Grade 0	Grade 1	Grade 2	Grade 3
Acute	11/44	23/44	10/44	0/44	17/44	18/44	9/44	0/44
	25%	52.27%	22.72%	0%	38.63%	40.90%	20.45%	0%
At 1 month	32/43	1/43	1/43	0/43	36/43	7/43	1/43	0/43
	74.41%	2.30%	2.30%	0%	83.72%	16.28%	2.30%	0%
At 6 months	23/28	5/28	0/28	0/28	22/28	5/28	1/28	0/28
	82.14%	17.85%	0%	0%	78.57%	17.86%	3.57%	0%
At 12 months	16/20	4/20	0/20	0/20	13/20	6/20	1/20	0/20
	80%	20%	0%	0%	65%	30%	5%	0%

### Predictors of toxicity

Regarding the small number of grade 2 toxicity events, we grouped the patients into without (grade 0) or with toxicity (grade 1 or 2). Any dose-volume variable expressed as a percentage was correlated with toxicity; nevertheless, some of them were correlated when expressed as absolute volume (cubic centimeter). The probability of having acute intestinal toxicity was statistically associated to the volume of rectum receiving ≥28 Gy (*p* = 0.029). In our sample, beyond 15 cm^3^, the probability of having acute intestinal toxicity is over 60% (test efficacy 0.7045). Fifteen of the 45 patients had a rectal volume of >15 cm^3^ receiving that dose. In contrast, only five patients did not meet the dose limit actually used in the treatment planning (the percentage of rectal volume receiving ≥28 Gy should be less than 40%). In our trial, the age was a predictor of GI toxicity after a month of treatment (*p* = 0.0005). Under 72 years old, the probability of having GI toxicity is below 12% (test efficacy 0.63). No correlation was found between urinary toxicity and the other analyzed variables.

### Quality of life

**Figure 2:**
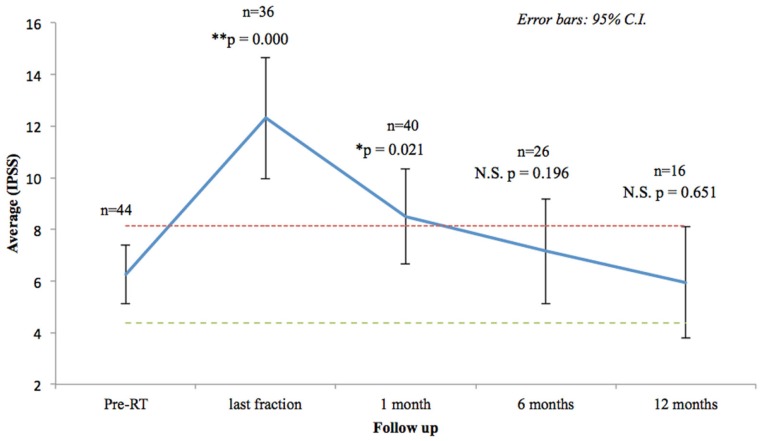
**Urinary quality of life (IPSS questions 1–7)**. The scores range from 0 to 35 with higher values representing worsening urinary symptoms. Numbers above each time point indicate the number of observations contributing to the average. The thresholds for minimally important clinical differences (MID) are marked with dashed lines.

**Figure 3:**
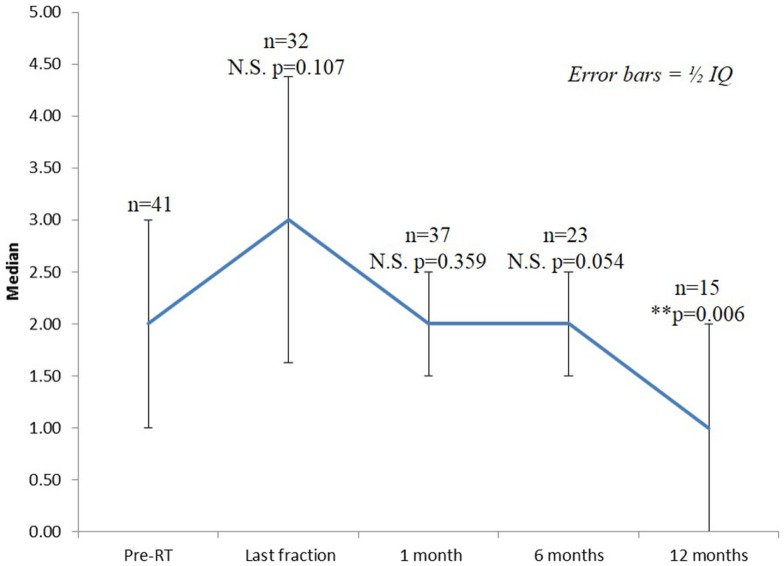
**Urinary quality of life (IPSS question 8)**. The score range from 0 to 6 with higher values representing the patient is more dissatisfied with his current urinary function.

Seventy-three percentage of the patients had mild to moderate lower urinary tract symptoms prior to SBRT with a mean initial IPSS of 6.25 (range, 0–14, SD 3.75) (Figure 2). That score increased significantly at the time of the last fraction (*p* = 0.000) and at 1-month follow-up (*p* = 0.021), returning to baseline at 6 months (*p* = 0.196). One month post-treatment, however, mean IPSS was barely above the MID (MID level 8.125). Urinary-related quality of life (Figure 3), derived from the Q8 score, worsened not significantly during radiotherapy (*p* = 0.107) returning to the baseline levels at 1 month after the treatment. At 12 months follow-up patient’s perception of their urinary function improved significantly (*p* = 0.006) compared with baseline.

## Discussion

Presently, a handful of prospective studies using SBRT for prostate cancer have been published, which have shown successful outcomes with low toxicity profiles (2–8, 13–17). In an attempt to overcome the problem of the small number of patients included in each study, a consortium was formed to pool the available data of phase II prospective trials from eight institutions that used Cyberknife (9). A total of 1100 patient were enrolled with a median follow-up of 36 months. The 5-year biochemical relapse free survival rate was 95, 84, and 81% for low-, intermediate-, and high-risk patients, respectively, supporting that Cyberknife SBRT can be considered as an appropriate therapeutic option for selected patients. However, there is a limited experience on prostatic SBRT delivered by conventional linear accelerator or helical tomotherapy. Emerging data from single institutional series suggest that this approach can achieve similar low rates of grade ≥3 early toxicity compared with robotic SBRT (Table 4). Conventional LINACs protocols usually use a more protracted overall treatment time (alternate days or weekly fractions), reflecting the concern about acute tolerance and consequential late effects. In a series of 64 patients who received five fractions of 7.25 Gy, every-other-day treatments resulted in substantially less frequent grade 1–2 urinary toxicity (17 vs. 56%, *p* = 0.007) and less frequent grade 1–2 rectal toxicity (5 vs. 44%, *p* = 0.001) compared with daily treatments (10). Our study was designed to administer the treatment fractions every-other-day over 2.5 weeks, while delivering to the late-responding normal tissues the same biologically equivalent dose as the standard 20-fraction hypofractionated scheme used in our institution. In order to obtain a homogeneous dose distribution, avoiding hot spots over 105% in the periurethral zone of the prostate, the full dose was prescribed to 95% of PTV, instead of 70–80% isodose usually used in HDR-like SBRT. With the aim of reducing urinary symptoms, the Georgetown University Hospital changed his Cyberknife SBRT protocol to limit the prescription isodose from ≥70 to ≥80% and the maximum prostatic urethral dose from 133 to 110%. In their opinion, the relatively high incidence of acute urinary grade 3 observed in their series was caused by the highly heterogeneous dose distribution inside the prostate, with hot spots up to 40% above the prescribed dose, and also by the patient selection, as they treated the patients not suitable for brachytherapy (large volume, bad flow, high IPSS, TURP, or abdominal surgery, hip joint prostheses) (2).

**Table 4:** **Acute toxicity SBRT in Cyberknife and non-robotic series**.

	GU grade 2 (%)	GU grade 3 (%)	GI grade 2 (%)	GI grade 3 (%)	Scheme
**CYBERKNIFE**					
Aluwini et al. (2)	15	8	12	2	38 Gy/4 consecutive fx (SIB 44 Gy/4 fx in DIL)
Bolzicco et al. (3)	12	0	18	0	35 Gy/5 consecutive fx
Friedland et al. (4)	–	0.9	–	0.9	35–36 Gy/5 consecutive fx
Kang et al. (5)	13.6	0	9.1	0	32–36 Gy/4 consecutive fx
Katz et al. (6)	4–4.7	0	3.6–4	0	35–36.25 Gy/5 consecutive fx
Oliai et al. (7)	19	4	4	0	36.25–37.5 Gy/5 consecutive fx
**NON-ROBOTIC SBRT^a^**					
Alongi et al. (13)	40	0	10	0	35 Gy/5 fx on alternate days
Boike et al. (14)	7–33	0	0–27	0	45–50 Gy/5 fx separated ≥36 h
Loblaw et al. (15)	19	1	10	0	35 Gy/5 fx once a week
Madsen et al. (16)	21	2	13	0	33.5 Gy/5 consecutive fx
Menkarios et al. (17)	31	5	14	0	45 Gy/9 fx once a week
Current study	22.7	0	20.4	0	43.84–45.20 Gy/8 fx on alternate days

*^a^Radiotherapy technique: volumetric modulated arc therapy (13), step and shoot IMRT (14–16), 3DCRT (17), helical tomotherapy (current study)*.

*SIB, simultaneous integrated boost; DIL, dominant intraprostatic lesion*.

Inter-fraction changes in prostate shape are not uniform throughout the length of the gland. A distended rectum can lead to an anteroposterior shift in the cranial aspect of the gland and not in the apex. As a result, it is sometimes the case that a correction compromises the adequate coverage of the anterior part of the CTV. To avoid this, the PTV has been expanded anteriorly to the pubic bone. This, together with the overestimation of prostate gland volumes using traditional CT-based planning, since MRI was only used for prostate cancer staging, results in larger CTV/PTV in our series compared with Cyberknife studies.

A limitation of the study is the lack of assessment and management of intrafraction motion. The deviation of prostate intrafraction motion distribution is a function of stool/gas volume. While the effectiveness of dietary intervention, oral laxatives and rectal enema is controversial (25–29), intrafraction motion is significantly reduced by endorectal balloons (30), specially with longer treatment times. An adequate patient preparation protocol before treatment and the daily use of endorectal balloon can effectively stabilized prostate motion for 90% of the fractions using a 3-mm internal margin (31). Additionally, patients treated with a water-filled endorectal balloon reported significantly less urgency and incontinence, while their treatment plans showed significantly lower doses to the anal wall, rectal wall and all pelvic floor muscles (32). In our institution the inter-fraction prostate displacement is taken into account by MVCT images with soft tissue registration without fiducials. With manual registration of planning CT and kV cone-beam CT (CBCT) without implanted markers the inter-observer variability results in errors of 2–3 mm (33). CBCT with implanted markers can decrease inter-observer variability within 2 mm compared with soft tissue alignment (34). So, consideration needs to be given to margin design at each institution when using soft tissue matching due to the above described increased inter-observer variability.

We observed that the urinary function, measured on the AUA/IPSS scale, recovered as in other non-robotic (16) and Cyberknife trials (35–37). The median AUA score increased slightly at the 1-month follow-up and then returned to baseline values at 3-month and subsequent follow-up intervals. Madsen et al. (16) also found that after 12 months of follow-up, more than a half of the patients reported improvement of their scores compared to baseline. King et al. reported quality of life prospectively measured among 864 patients from phase II clinical trials of SBRT (11). For urinary QOL, a significant but modest decline was also most notable within the first 3 months, which had mostly recovered by 6 months, remained stable thereafter. For those patients with poorer urinary function at baseline (i.e., worst 25th or fifth percentile), a gradual improvement in urinary QOL was in fact observed beginning 6 months after treatment and progressing to better than baseline function over the 6-years follow-up. No differences were seen with the addition of AD or as a function of patient age. A similar trend was seen for bowel QOL.

Low evidence is available on the predictors for acute toxicity after prostate SBRT. We observed that the volume of rectum receiving ≥28 Gy is correlated with acute intestinal toxicity. Kim et al. enrolled 91 patients on a dose-escalation (from 45 to 50 Gy in five fractions) phase I/II study (38). According to our results, they observed that grade ≥2 acute rectal toxicity was significantly correlated with more than 50% of the rectal wall receiving ≥24 Gy. In their opinion, minimal vascular/stromal injury is likely to occur at that dose level, allowing an adequate blood supply to the recruited stem cells leading to an effective repair of the injury, provided that less than half the rectal wall receives ≥24 Gy. We have observed that the volume of rectum receiving a particular radiation dose was an independent predictor of rectal toxicity when using actual volume rather than percent volume. Others authors have had the same findings and are then currently using actual rectal volume when setting up planning constraints (39).

In our series, 71% of the patients are intermediate-high risk. Most SBRT trials have only included low-risk cases due to the concern that the tight margins required to limit the normal tissue doses may not be adequate to treat the microscopic disease. Ju et al. (37) reported that for the majority of their patients, treated with Cyberknife SBRT, the coverage of the 33-Gy isodose line (76 Gy EQD2, α/β 1.5) is more than 5 mm beyond the prostate, excluding posterior direction, where it is ≤3 mm in most cases. In our study, one half of the seminal vesicles were included within the CTV, and the PTV extended at least 5 mm from prostate gland. In any patient, more than 95% of the PTV received the 98% of the prescribed dose (equivalent to 92 Gy EQD2, α/β 1.5). We therefore believe that our approach should effectively eradicate the microscopic extraprostatic disease in high-risk prostate cancer patients.

## Conclusion

Our scheme of eight fractions on alternate days (EQD2 = 87–92 Gy, α/β 1.5) delivered using helical tomotherapy is well tolerated. We observe an impact on urinary quality of life at the end of the radiotherapy treatment and 1 month later, with subsequent recovery to baseline at 3 months follow-up. In order to decrease acute toxicity, we recommend 15 cm^3^ as the cut-off of the rectal volume not to exceed 28 Gy (EQD2 = 48.4 Gy, α/β 3). However, the rectal volume receiving that dose, expressed as a percentage, was not correlated with the intestinal toxicity. That supports using actual volume rather than percent volume in the treatment planning. The use of endorectal balloon catheters would help us to reduce intrafraction movement, reduce moderate doses in lateral and posterior rectal walls and to determine the position of the anterior rectal wall at daily MVCT. A comparison between this 8-fraction series and our contemporary 20-fraction series, with total doses equivalent for late-responding tissues, is ongoing.

## Conflict of Interest Statement

The authors declare that the research was conducted in the absence of any commercial or financial relationships that could be construed as a potential conflict of interest.
